# The Development of an Electronic Clinical Decision and Support System to Improve the Quality of Antenatal Care in Rural Tanzania: Lessons Learned Using Intervention Mapping

**DOI:** 10.3389/fpubh.2021.645521

**Published:** 2021-05-20

**Authors:** Sandra van Pelt, Karlijn Massar, Laura Shields-Zeeman, John B. F. de Wit, Lisette van der Eem, Athanas S. Lughata, Robert A. C. Ruiter

**Affiliations:** ^1^Department of Work and Social Psychology, Maastricht University, Maastricht, Netherlands; ^2^Department of Mental Health and Prevention, Netherlands Institute for Mental Health and Addiction, Utrecht, Netherlands; ^3^Department of Interdisciplinary Social Science, International Public Health, Utrecht University, Utrecht, Netherlands; ^4^Woman Centered Care Project, A Project of the African Woman Foundation, Magu, Tanzania

**Keywords:** maternal health, antenatal care, digital health, electronic clinical decision and support system, intervention mapping, healthcare worker performance, implementation

## Abstract

It is widely recognised that high quality antenatal care is a key element in maternal healthcare. Tanzania has a very high maternal mortality ratio of 524 maternal deaths per 100,000 live births. Most maternal deaths are due to preventable causes that can be detected during pregnancy, and antenatal care therefore plays an important role in reducing maternal morbidity and mortality. Unfortunately, quality of antenatal care in Tanzania is low: Research has shown that healthcare workers show poor adherence to antenatal care guidelines, and the majority of pregnant women miss essential services. Digital health tools might improve the performance of healthcare workers and contribute to improving the quality of antenatal care. To this end, an electronic clinical decision and support system (the Nurse Assistant App) was developed and implemented in Tanzania in 2016 to provide digital assistance during antenatal care consultations to healthcare workers. The current study systematically evaluated the development and implementation process of the Nurse Assistant App in Magu District, Tanzania, with the aim of informing future programme planners about relevant steps in the development of a digital health intervention. Desk research was combined with semi-structured interviews to appraise the development process of the digital health tool. We employed the criteria stipulated by Godin et al., which are based on the six steps of Intervention Mapping [IM; Bartholomew Eldredge et al.]. Findings indicated that five of the six steps of IM were completed during the development and implementation of the Nurse Assistant App. Tasks related to community engagement, adjustment to local context, implementation in the practical context in collaboration with local partners, and rigorous evaluation were accomplished. However, tasks related to identifying theory-based behaviour change methods were not accomplished. Based on the lessons learned during the process of developing and implementing the Nurse Assistant App, we conclude that programme developers are recommended to (1) engage the community and listen to their insights, (2), focus on clear programme goals and the desired change, (3), consult or involve a behaviour change specialist, and (4), anticipate potential problems in unexpected circumstances.

## Introduction

Current best estimates from the United Nations show that one of the countries with a “very high” maternal mortality ratio is Tanzania, with 524 maternal deaths per 100,000 live births (1). The consequences of maternal death are far-reaching, as it impacts women as well as their children, families, and communities (2). The death of a mother negatively influences schooling and nutrition for children, results in economic loss, and increases the chances of death for her baby (2–4). Most maternal deaths are due to preventable causes that can be detected during pregnancy, such as haemorrhage, infection, unsafe abortion, hypertensive disorder, and obstructed labour (5). Therefore, antenatal care (ANC) is a crucial intervention to promote maternal and child health (6–9) and identify issues early during pregnancy. ANC improves the survival and health of mothers through the prevention and management of pregnancy-related complications and by providing an entry point for health contact with pregnant women (8, 10). In addition, ANC offers an opportunity to promote facility-based deliveries, which is considered as the key intervention for reducing maternal mortality in low- and middle-income countries (6, 11–15).

It is widely recognised that the quality of care in Tanzania needs to be improved since the majority of pregnant women miss essential services (15–18). A nationwide survey found that 47% of the women receiving ANC were not informed by the healthcare workers about danger signs (19), 29% had no blood pressure taken and 40% had no urine sample taken during the entire pregnancy (20). These results have been confirmed by multiple studies conducted in several districts of Tanzania (16, 17, 21–25). It has been shown that ~20% of severe maternal morbidity could be avoided by improving the quality of ANC, including more rigorous prevention and management of severe anaemia and the early detection and management of hypertensive disorders in pregnancy (16). Despite the commitment of the Tanzanian Ministry of Health, Community Development, Gender, Elderly and Children to prioritise maternal, new-born and child health by enhancing ANC services, reducing maternal death remains a challenge (15).

One innovative solution to improve the quality of ANC might be an electronic clinical decision and support system for use by healthcare workers during ANC, as it may have the potential to guide and improve healthcare workers' performance and adherence to guidelines (26–29). A study conducted in Tanzania on the use of an electronic clinical decision and support system to improve HIV care, found high acceptability of healthcare workers to use the device (30). In an effort to contribute to this solution, the Woman Centered Care Project – a project run by the African Woman Foundation between 2013 and 2017 to improve maternal health outcomes – conducted a needs assessment in Magu district, Tanzania. The aim of the needs assessment was to explore healthcare workers' and pregnant women's perceptions regarding ANC and the use of an electronic clinical decision and support system during ANC (31)^1^.

The needs assessment undertaken through the Women Centered Care Project consisted of semi-structured in-depth interviews with 16 healthcare workers and 19 pregnant women recruited from a sample of healthcare facilities in Magu district (31)^1^. Both healthcare workers and pregnant women expressed a positive attitude toward ANC and acknowledged its importance. However, they also expressed a need for improved ANC care delivery, particularly availability of diagnostic tests, and strategies to improve performance and strengthen motivation of healthcare workers to provide antenatal care (31)^1^. Healthcare workers explained that differences in healthcare worker's provision of antenatal care were due to differences in level of education, with some workers lacking experience, knowledge, and skills (31). While the pregnant women were grateful for the care received, one-third of the participants felt that healthcare workers should perform better and questioned healthcare workers' willingness to perform or explain certain procedures^1^. Regarding the use of an electronic clinical decision and support system during ANC, the interviews found positive views among both healthcare workers and pregnant women (31)^1^. Both expressed that an electronic clinical decision and support system could improve the quality of ANC due to improved record-keeping, improved performance of healthcare workers, and improved communication between healthcare workers and pregnant women. Healthcare workers expressed that an electronic clinical decision and support system could improve their performance because the structured guidance it offers would make it easier to ask additional questions, detect high-risk pregnancies, and know what to do in case of any complications (31).

**Figure 1:**
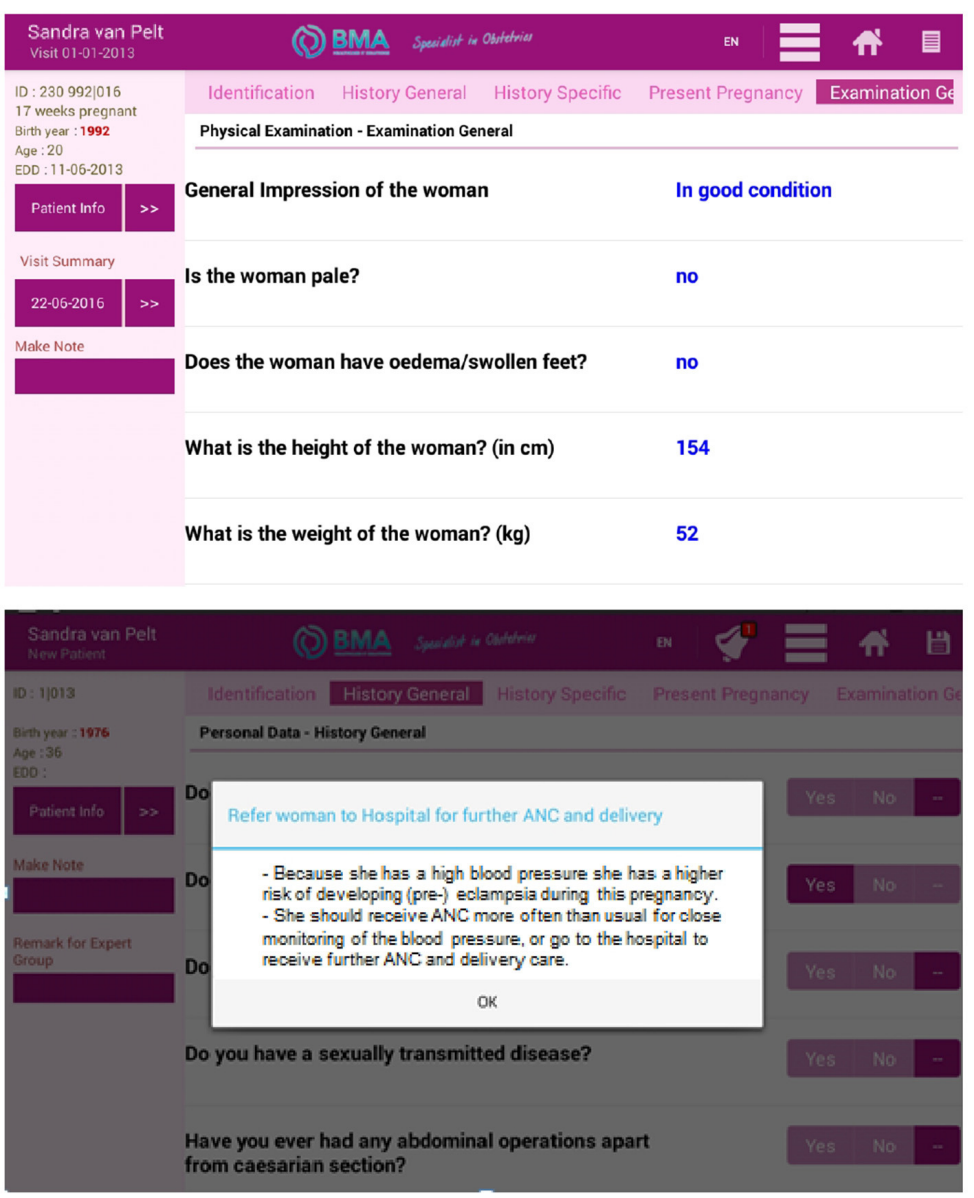
Example of one of the sections of the Nurse Assistant App.

Guided by these findings of the needs assessment, a Nurse Assistant App (NAA) was developed as part of the Women Centered Care Project. The NAA is a tablet-based application to improve the quality of ANC by strengthening healthcare workers' performance. Specifically, the NAA consisted of a comprehensive questionnaire to be filled in by a healthcare worker during the ANC consultation, based on results obtained through observations and/or responses of the pregnant woman. The NAA offers a step-by-step guide through the essential interventions of an ANC visit, categorised into history taking, physical examination, laboratory tests, medication provision, and health education. Abnormalities in any of these sections were signalled to the healthcare worker through automatically generated “alarm bells.” After completing all steps, the NAA generated a clinical summary and provided the healthcare worker with advice on treatment, referral, and follow-up according to national and international evidence-based guidelines. For an example of one of the sections of the NAA, see Figure 1.

The NAA was developed by professionals in practise, and at the time of its development in 2014, little was known about the process of successfully developing and implementing digital health interventions in low- and middle-income countries. To our knowledge, this knowledge gap persists and available evidence focuses on the outcomes of digital health interventions and much less on process optimization in the development or implementation phase (32, 33), which is critical to a digital tool having an impact on health outcomes. Evaluating the development process of a digital health intervention such as the NAA allows for identification of lessons learned to inform future programme planners in low- and middle-income countries on important considerations and pitfalls to avoid when developing a digital health intervention.

This paper evaluates the development and implementation process of the NAA, using Intervention Mapping (34) as a guide to assess whether a systematic and theory- and evidence-based approach was employed in the development of the digital tool. In addition, models and matrices, which are essential parts of Intervention Mapping, where retrospectively created to evaluate the scientific base of the choices made during the development and implementation process of the NAA. The findings of this study will help to inform future programme planners in low- and middle-income countries developing a digital health intervention as well as contribute to a greater understanding of factors that can help a tool like the NAA to be meaningful for end-users and be sustained in practise after the pilot phase.

## Methods

### Design

This qualitative study draws upon two sources of data: documents describing the development and implementation process of the NAA, complemented by semi-structured interviews designed to verify the results of the desk research.

### Evaluation Framework

**Figure 2:**
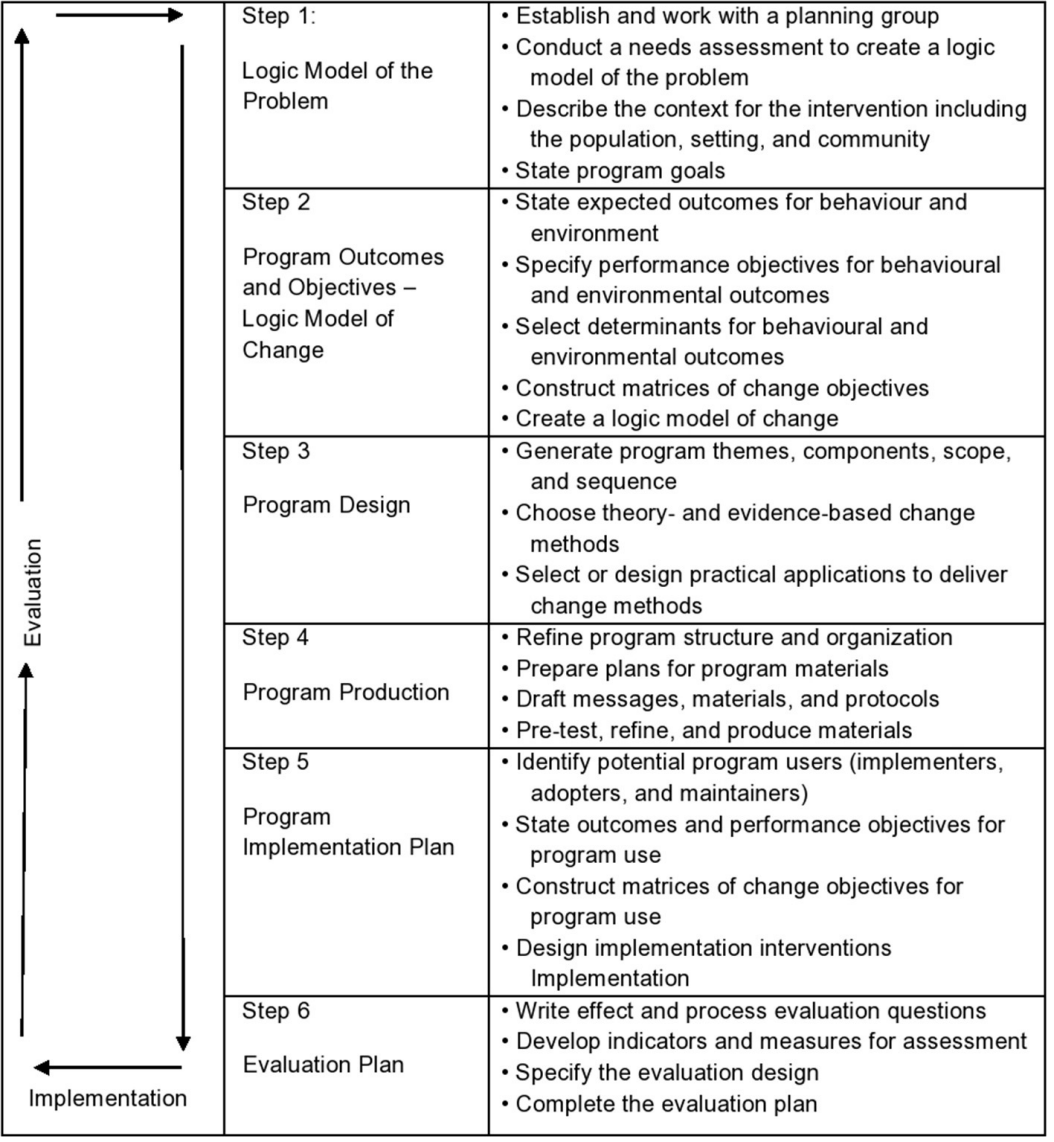
The six steps of Intervention Mapping.

This evaluation is guided by Intervention Mapping, a six-step protocol for theory- and evidence-based intervention development that can also be used for the *post-hoc* evaluation of the soundness of digital health interventions, such as an electronic clinical decision and support system (35). Intervention Mapping provides systematic guidance to programme developers that facilitates making sound decisions during the process of intervention development (34). In step 1 of Intervention Mapping, the health problem is analysed through a needs assessment and the subsequent development of a logic model of the problem to specify the causes and contributing factors related to the health problem. Step 2 focusses on what behaviour and underlying personal determinants need to change in order to reduce the health problem, visualised in a logic model of change. In step 3, an intervention is designed, based on behaviour change theory and relevant change methods. In step 4, intervention materials, activities, and protocols are designed and pre-tested and the final intervention is produced. In step 5 an implementation plan is developed to ensure adoption and full and correct use of the intervention and aim for long-term use. Step 6 focusses on programme evaluation including both an effect and process evaluation. All six steps consist of distinct tasks that need to be completed before proceeding to the next step. At the same time, Intervention Mapping allows for an iterative work process going forwards and backwards through steps and tasks. A flowchart of the different steps is shown in Figure 2.

To assess the soundness of the development process of the NAA, we make use of the planning evaluation tool developed by Godin et al. (35), which is based on the six steps of Intervention Mapping (34). The planning tool was developed to assist professionals in performing a rigorous evaluation of an intervention and consists of 40 criteria that make up 19 tasks (see Table 1) that can be scored. The application of this tool to the intervention development process provides insight into the steps and tasks that were undertaken and completed during the development and implementation of the NAA. To score the criteria of step 1 and step 2 in the planning tool, models, and matrices were retrospectively created. This gave insight into the extent to which choices made by the project team were in line with models that would have been created based on the available empirical literature, theory and collected data. The logic model of the problem was retrospectively created mainly based on the results of the needs assessment while the logic model of the problem was retrospectively created based on stakeholder meeting reports. Previous research has been published documenting a similar method of retrospectively evaluating an intervention (35–37).

**Table 1:** Planning evaluation tool of Godin et al. (35).

**Criteria**	**Intervention Mapping task**	**Accomplishment**	**Step completed**
**Step 1 logic model of the problem**			Yes
	**Task 1 Identify the problem**		
1	Consult literature	+	
2	Validate with local supporters	+	
	**Task 2 Identify the target population**		
3	Socio-demographic profile	+	
4	Socio-cultural context	+	
	**Task 3 Identify determinants**		
5	Consult literature	+/–	
6	Gather information on the population	+/–	
	**Task 4 Analyse the environment**		
7	Identify places, methods and times to contact the participants	+	
8	Identify hindering and facilitating factors	+	
9	Identify partners and their respective roles	+	
**Step 2 logic model of change**			No
	**Task 5 Specify the population**		
10	Consider the particularities	+	
	**Task 6 Overall objective**		
11	Word precisely the selected change	+/–	
	**Task 7 Performance objectives**		
12	Specify what should be obtained	**–**	
13	Develop objectives based on theory, empirical data or Deep Understanding validate with partners	**–**	
14		**–**	
	**Task 8 Choice of determinants**		
15	Choose with respect to their connection with the target behaviour	**–**	
16	Choose with respect to their potential success	**–**	
17	Validate with partners	**–**	
	**Task 9 Change objectives**		
18	Related to performance objectives and determinants	**–**	
19	Based on theoretical notions	**–**	
20	Validate with partners	+/–	
**Step 3 programme design**			Yes
	**Task 10 Choose the models**		
21	Support with tested theoretical methods	**–**	
22	Consider population characteristics (determinants)	+/–	
	**Task 11 Translate into strategies**		
23	Support with theory	**–**	
24	Validate with partners	+	
**Step 4 programme production**			Yes
	**Task 12 Organisational structure**		
25	Consider limitations of the milieu	+/–	
26	Carry out with partners	+	
27	Train and support workers	+	
	**Task 13 Sequence and content of activities**		
28	Activities related to objectives	+	
29	Realistic calendar	+	
30	Validate with partners	+	
	**Task 14 Production of material**		
31	Involvement of partners	+	
32	Begin scheduled activities	+	
33	Accessible and properly communicated	+	
34	Adapt the material	+	
**Step 5 programme implementation**			Yes
	**Task 15 Support of decision-makers and community**		
35	Active partners	+/–	
36	Identify the person in charge	+	
**Step 6 evaluation plan**			Yes
	**Task 16 Evaluation plan**		
37	Plan before implementation	+	
	**Task 17 Process**		
38	Document information about the population and the intervention	+	
	**Task 18 Impact**		
39	Measure the degree to which objectives are archived	+	
	**Task 19 Communication**		
40	Discuss findings with partners	+/–	

### The NAA Project Team

In 2012, the African Women Foundation created a partnership with Crop Marketing Bureau (CROMABU), a Tanzanian organisation aimed at empowering small-scale farmers by using Information and Communication Technologies (ICT) in Magu district, Tanzania and commenced conducting research related to maternal health. In 2013, the Women Centered Care Project was initiated. The project team of the Women Centered Care Project consisted of one Tanzanian head of staff, one Dutch programme manager, one Dutch research officer, one Dutch liaison programme officer, and three Tanzanian research assistants. The research activities of the project were conducted in close collaboration with four universities (three in the Netherlands and one in Tanzania). The programme manager worked closely together with the board of the African Woman Foundation in the Netherlands, which had an advisory, fund-raising, partnership, and decision-making role. The programme manager also worked on the development and maintenance of the NAA in collaboration with ICT Healthcare Technology Solutions (formerly Buro Medische Automatisering B.V.), a Dutch private company specialised in digital solutions in the domain of obstetrics (38).

### Desk Research

To assess processes and milestones in the development and implementation of the NAA, desk research was conducted using digital project archives, project documents, and content from the NAA itself. Data sources consisted of minutes of meetings, for which a template was made available at the time (i.e., minutes of 67 bi-weekly project team meetings from November 2013 until August 2016, minutes of meetings with stakeholders, including three meetings with the district reproductive and child health coordinator, minutes of 13 meetings with healthcare workers at their health facility, and minutes of 59 meetings with district officials held twice a year per subdivision of the district); reports on project activities that were approved by the board of the African Woman Foundation (i.e., workshop and training sessions offered by the project team to healthcare workers, monthly financial reports of the project, annual and quarterly project reports); field notes (i.e., supervision visits undertaken by the project team to all participating healthcare facilities, logs of the technical support provided by the research assistants of the project team to healthcare workers working with the NAA); personal timesheets of members of the project team kept for verification of working hours; and e-mail conversation between board members, the project team, and the ICT specialists. One researcher conducted the search among the data sources and carried out the document synthesis, which was verified by a second researcher. Project-team discussions were held to discuss comprehensiveness of data, access to archives, and making sense of the data.

### Data Analysis

Data obtained through the desk research were used to assess the development and implementation process of the NAA by scoring the criteria specified in the planning tool. Firstly, the two researchers worked independently from each other to score each of the 40 criteria of the planning tool (+ fully accomplished; +/− partially accomplished; – not accomplished). Secondly, the two researchers jointly evaluated whether or not a task was accomplished. Tasks were considered accomplished if at least one criterion was coded as “fully accomplished” (35). Thirdly, the two researchers jointly evaluated whether each of the Intervention Mapping steps was completed. Steps were considered completed when at least half of the tasks within that step were scored as accomplished (35).

For example, step 3 of the Intervention Mapping approach consists of two tasks, reflected in four criteria of the planning tool (numbered 21–24). If, for example, criterion 21 was scored as “not accomplished” and criterion 22 as “partly accomplished,” task 10 was considered to be not accomplished overall. However, if criterion 24 was scored as “accomplished” and criterion 23 as “not accomplished,” task 11 was considered accomplished. Consequently, since half of the tasks in step 3 were accomplished, the step was considered completed (see Table 1).

### Semi-structured Interviews

To complement the results of the analysis, semi-structured interviews were held with two developers of the NAA selected to gain insight into the development process from distinct perspectives. The team of the Women Centered Care Project consisted of six people. Of these, only two could be contacted, as others had moved on: a Tanzanian project research assistant based in Tanzania and a Dutch programme researcher based in Magu district, Tanzania at the time of NAA development. Both these participants were involved from the beginning to the end of the development of the NAA, and had the possibility to influence the decision making process regarding the development of the NAA. It proved not possible to include software developers involved in the development of the NAA, as both had moved on. Participants were approached individually by e-mail with a request for participation and both agreed. During the semi-structured interviews, participants were encouraged to share their critique on the completed planning tool, to verify the results and check for any inconsistencies. Prior to the interview, the completed planning tool was sent to the participants so that they could prepare in advance. Interviews were conducted by phone and lasted for ~40 min. Interviews were audio-recorded with verbal informed consent of the participants. During the interview, the intervention planning tool was discussed until consensus was reached by the interviewee and the interviewer about the appropriate coding.

### Practical Execution of the Six Steps of Intervention Mapping

Data obtained through the desk research and semi-structured interviews that did not correspond to criteria of the planning tool were included as additional information to assess the practical execution of the development and implementation of the NAA. Although, the analysis of the practical execution of the six steps of Intervention Mapping is not officially part of the planning tool, we believe that the examples on the practical execution of the six steps might help to inform future programme planners. Intervention Mapping was retrospectively used to structure the data and assess what steps of Intervention Mapping were executed. Specifically, required models and matrices were retrospectively created based on the data. At the time of the development of the NAA, Intervention Mapping was not applied, therefore this part of the data created a comprehensive overview of all activities conducted during the process of development and implementation of the NAA.

## Results

A description of the completed planning tool combined with the analysis of the practical execution of the six steps of Intervention Mapping is presented below to evaluate the development and implementation of the NAA. Results will be described following the six steps of Intervention Mapping.

### Step 1: Logic Model of the Problem

Research regarding the needs assessment was performed in 2015 and 2016 by the programme researcher in collaboration with the four universities and the local government in Magu district. As described in the introduction, the result of the needs assessment, combined with literature on maternal health issues in rural Tanzania, provided a clear description of the problem the community was facing as well as of the target population of the intervention. Several focus group discussions, workshops, and technical meetings were conducted to discuss the findings of the needs assessments. Larger groups were brought together when the focus was more on brainstorming or dissemination of key messages to a larger audience. Smaller group sessions facilitated the targeted exchange of ideas and the discussion of politically sensitive issues that may not be appropriate to address in a larger group setting.

**Figure 3:**
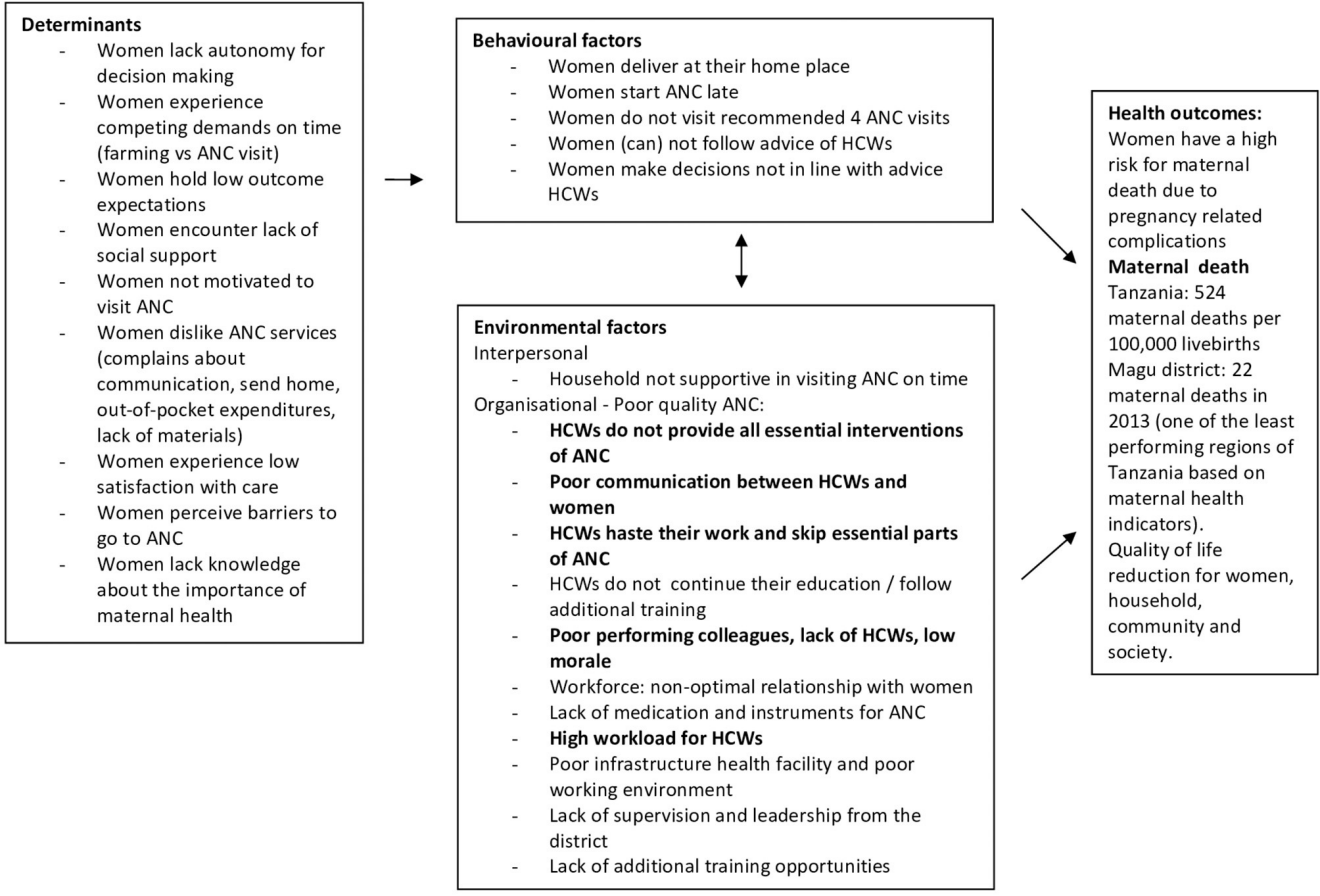
Retrospective logic model of the problem.

A logic model of the problem was created retrospectively, framed around the problem of high maternal mortality in Magu district, which was chosen as the health problem (Figure 3). The needs assessment highlighted that pregnant women did not receive ANC of sufficient quality, due to several behavioural and environmental factors. The behaviours of the healthcare workers became the main point of focus to reduce the high number of maternal death in the district, as visualised in Figure 3 in bold font. This focus was aligned with the a priori idea of the board of the African Woman Foundation in the Netherlands to develop a digital health tool for healthcare workers. The needs assessment showed that the highest burden of maternal health issues was in rural parts of the district. In rural areas, dispensaries are the main access point for reproductive health services, including antenatal care. It was specified that all healthcare workers in dispensaries were the target population for the intervention.

At the start of the Women Centered Care Project, a memorandum of understanding was signed by the local government, collaborating universities, and the African Woman Foundation to agree on a set of activities and division of responsibilities. From the start, it was a clear aim to share costs and responsibilities between the project team and the local government, to facilitate local ownership and sustainability. In practise, this meant that renovations of the healthcare facilities required to provide antenatal care were the responsibility of the local government, while costs related to the NAA intervention were covered by the Women Centered Care Project. On some occasions, there were discussions about tasks and responsibilities that reflected expectations of the local government that the Dutch project team would have the financial means to solve issues of health service infrastructure, for example to repair or replace a broken solar system.

In the planning tool, step 1 consists of 4 tasks which were all scored as accomplished. As a result step 1 was considered completed (see Table 1).

### Step 2: Logic Model of Change

**Figure 4:**
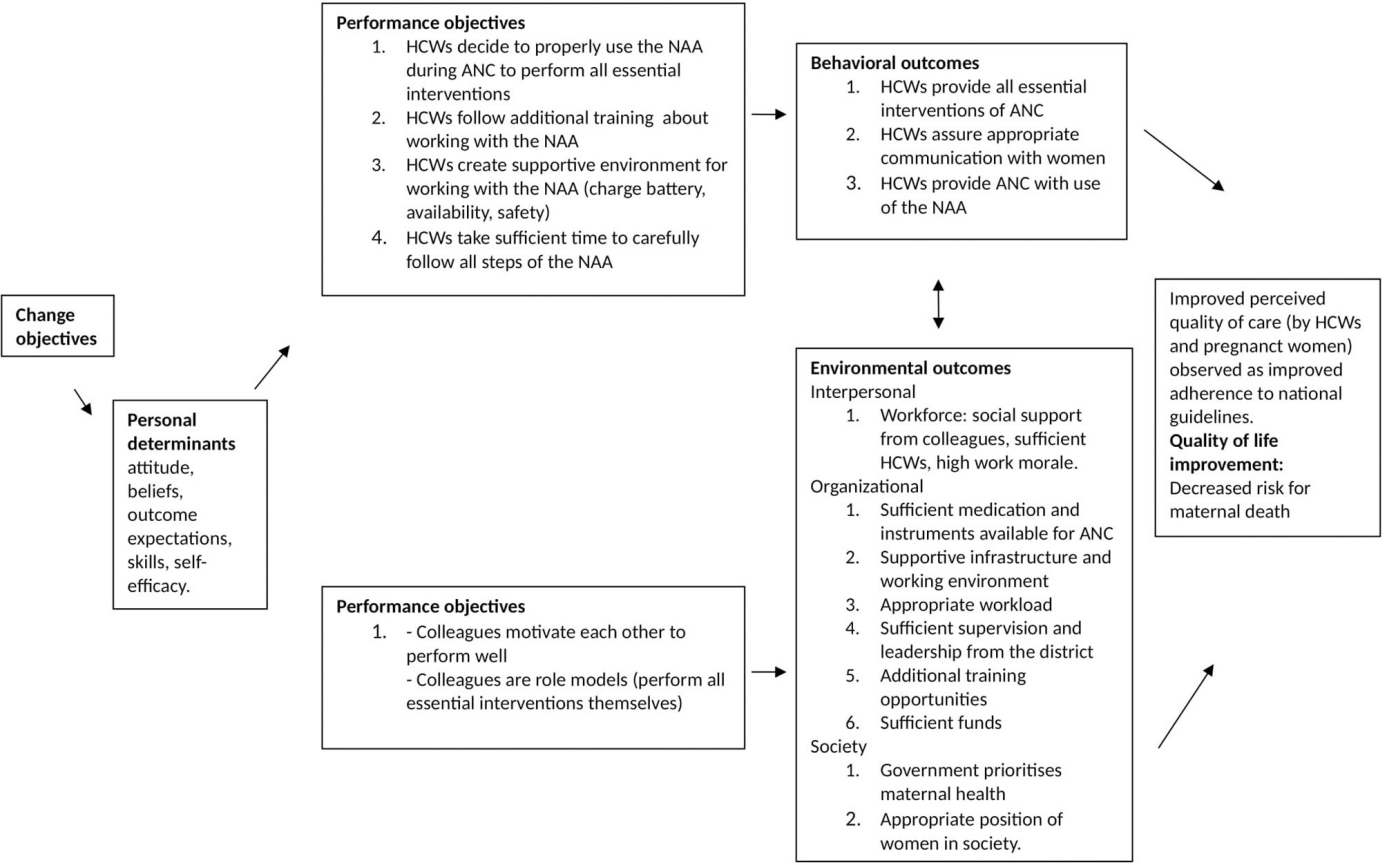
Retrospective logic model of change.

**Figure 5:**
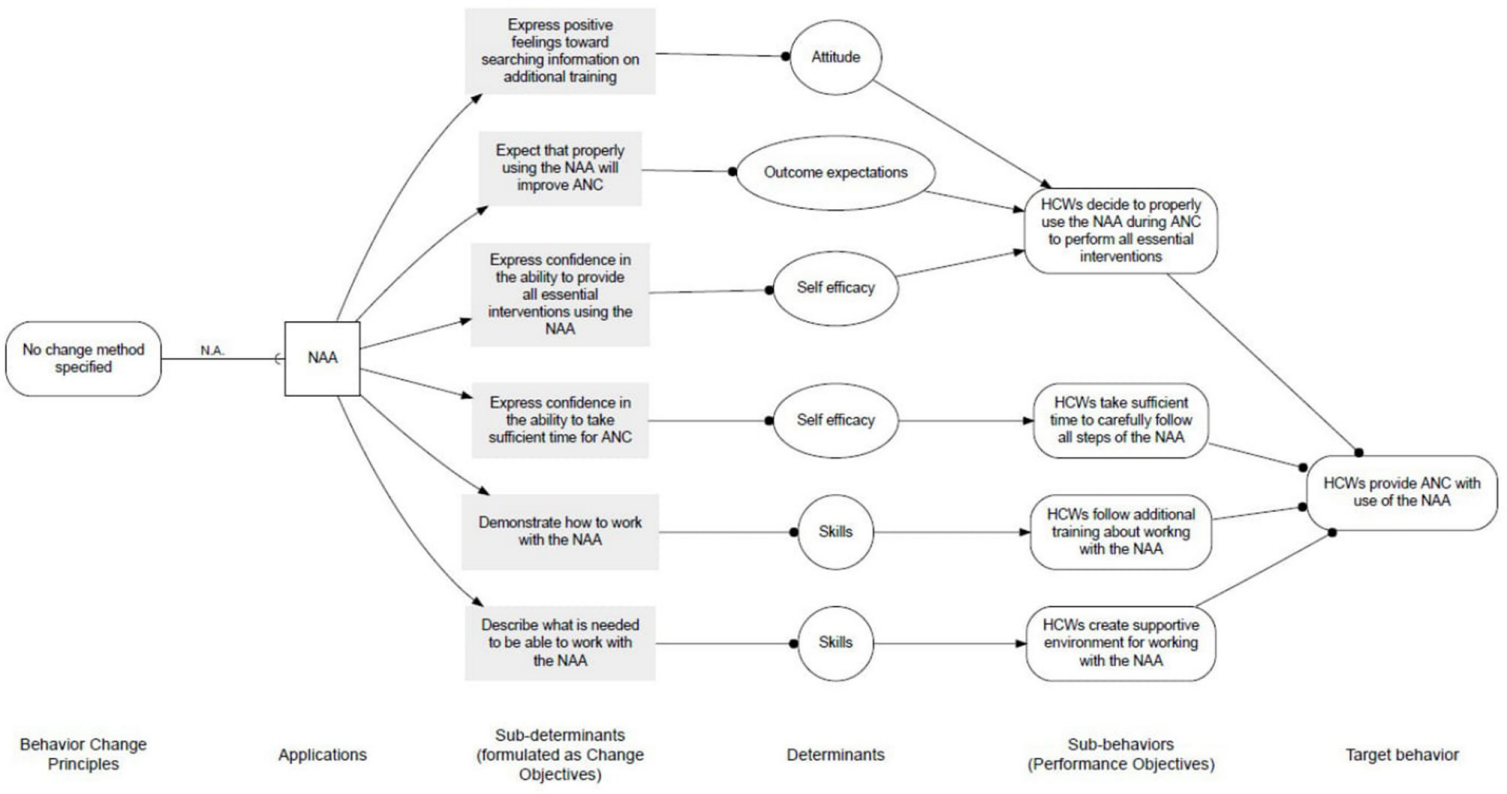
Behaviour change diagram.

The main objective of the project was to increase the quality of ANC by improving healthcare worker performance in rural dispensaries. The desired behavioural outcomes of the project were that healthcare workers would provide all essential ANC interventions and assure appropriate communication with pregnant women. The NAA would assist healthcare workers in conducting the ANC consultation in line with step-by-step guidance on effective ANC. Data of the desk research showed that the focus of the project team was hence on educating healthcare workers in appropriately providing ANC with the use of the NAA and assess change in healthcare workers' behaviour (and patient satisfaction) related to the implementation of the NAA. The retrospectively created logic model of change is visualised in Figure 4, and the related behaviour change diagram, including performance and change objectives, is shown in Figure 5. At the time of development of the NAA, these objectives were not specifically identified, and determinants for behaviour change were not explicitly taken into account. The objectives shown in Figure 5 were retrospectively created, based on data obtained from the desk research. As can be seen, at the level of behavioural determinants (change objectives) the project intended to increase the skills of healthcare workers regarding providing ANC with use of the NAA, increase their own outcome expectation that using the NAA will improve the quality of ANC, and influence attitudes and self-efficacy of healthcare workers toward the NAA positively.

The NAA was developed without making use of a theoretical framework for intervention development and therefore no behaviour change theories were specified to guide the design of the intervention to promote behaviour change among healthcare workers. The content of the NAA was based on national and international evidence-based guidelines that were verified by experts in practise, such as the local reproductive and child health coordinator and several local healthcare workers. These guidelines were also used to develop a scoring sheet for the evaluation of the performance of the healthcare workers. As can be seen in Table 1, no logic model for the development of the NAA was specified and no behaviour change theories were applied. Therefore, only one of the five tasks was accomplished and step 2 of Intervention Mapping was therefore considered incomplete.

### Step 3: Programme Design

The choice for the NAA as the best possible intervention to improve healthcare worker performance during ANC was based on reading empirical and theoretical literature on e-health interventions and maternal health in general. Although, this choice was made by the board of the African Woman Foundation in the Netherlands and could not be influenced by local stakeholders, the NAA programme itself - from initial draught until the final product – was developed in collaboration with all project team members, the local reproductive and child health coordinator, local healthcare workers, pregnant women receiving service at the participating sites, and the local government. All project team members were extensively involved and enthusiastic about the development of the NAA and contributed experiential knowledge to design and implement the intervention and fit appropriately in the local context. The NAA was presented and discussed extensively with all relevant stakeholders in the district to ensure cultural appropriateness of the NAA and obtain approval for its implementation.

Two criteria of step 3 of Intervention Mapping focus on community engagement, and were scored as (partly) accomplished; therefore only one task of step 3 was considered accomplished. Consequently, since half of the steps were considered accomplished, step 3 of Intervention Mapping was considered completed (see Table 1).

### Step 4: Programme Production

The choice for a tablet as the device to run the NAA application was made by the board of the African Woman Foundation in the Netherlands. This choice was made based on the practical features of a tablet: a portable device with a relatively large screen which makes it easy to make and send pictures and that does not require abundant pre-existing knowledge on mobile technology. A pre-pilot study was conducted in a different district, where healthcare workers trialled a test version of the NAA during ANC with the goal to assess user-friendliness, willingness to work with the NAA, and search for errors. The results of the pre-pilot study were used to further develop the NAA. The content of the NAA was translated from English to Kiswahili and vice versa during several rounds of forward and backward translation and verified for accuracy by local healthcare workers and an external Kiswahili language teacher. At several points in time, stakeholders were invited *via* official letters and had the opportunity to share their ideas on the NAA and make suggestions as to any modifications to the implementation process of the NAA, including the training activities. The final look, content, sequence, and structure of the NAA were created in close collaboration with local healthcare workers, pregnant women, the local reproductive and child health coordinator, the ICT specialists, and the local government, through several smaller or larger group meetings. The final version of the NAA was adjusted to the ANC work practise to minimise its effect on the existing workflow and make it an easy-to-use tool. To contribute to workplace adaptation, each healthcare facility received two tablets to enable running two separate ANC visits by two different healthcare workers at the same time.

In light of problems with the electricity and internet network in the district, all participating healthcare facilities were audited for the availability and stability of electricity and mobile network connections. It was ensured that at the start of the implementation of the NAA, all participating healthcare facilities were connected to the national electricity grid or had a good working solar power system. However, on some occasions, unforeseen challenges arose. Due to heavy rains, some healthcare facilities experienced more frequent and longer power interruptions than expected, and the relatively long time to fully charge the tablet in healthcare facilities using solar power was not anticipated. This resulted in occurrences of non-charged tablets and on some occasions, women did not receive ANC because healthcare workers did not want to proceed without the NAA. In addition, mobile network issues caused challenges as well since the files that needed to be sent to the NAA server were larger than expected. As a result, many electronic files of pregnant women that were not properly saved got lost and the pertinent information could not be re-entered during the specific ANC revisit. (In Tanzania, pregnant women are responsible for keeping their paper-based antenatal care card, which is used for documentation and contains the information relevant to their pregnancy. The implementation of the NAA did not influence this common practise to prevent losing medical files). Furthermore, one healthcare facility was not accessible during the rainy season due to bad road conditions. Step 4 of Intervention Mapping was considered completed, as all three tasks of this step were fully accomplished (see Table 1).

### Step 5: Programme Implementation

An implementation plan that systematically described the content and sequence of activities, was developed by the programme researcher in collaboration with the research team. The emphasis of the project on appropriate community involvement was reflected in ongoing activities that started already during the development phase. While the ownership of the NAA and responsibility for the implementation of the NAA remained with the project team of the Women Centered Care Project, healthcare workers and other stakeholders were consulted frequently.

The implementation plan consisted of several implementation strategies. Firstly, the project team provided training for healthcare workers about the essentials of ANC about 6 months before the introduction of the NAA in practise. During these training sessions, the initial concept of the NAA was also shared and input was asked regarding its development and implementation. Secondly, the reproductive and child health coordinators of each participating healthcare facility were invited for a workshop to view the test version of the NAA and share their ideas on improving the content. Thirdly, all healthcare workers providing ANC at participating facilities were invited for a 2-day NAA training to develop their skills needed to work with the NAA during ANC. Fourthly, participating healthcare facilities were visited by members of the project team to provide 3 days of on-the-job training. This on-the-job training was provided to continue guiding healthcare workers who needed extra support to incorporate the use of the NAA in ANC practise. Furthermore, a user manual was provided with the tablet, a technical support team was available for those healthcare workers who needed some extra support to work with the NAA or experienced technical difficulties, and all participating healthcare facilities were visited or called twice a month to ask if they experienced any difficulties.

Related to the implementation of the NAA in practise, some unforeseen challenges arose that needed to be solved. For example, three healthcare workers were not able to attend the 2-day NAA training and were therefore trained individually at their healthcare facility. Also, of the in total 15 tablets five broke down, of which three could not be replaced and some remaining “bugs” in the application were discovered. Healthcare workers did not always express their true opinions or would not call the technical support team when problems occurred. There was no script available in the implementation plan to guide responses to unexpected changes making it difficult to solve problems and ensure sustained use of the programme. Both criteria related to step 5 of Intervention Mapping planning tool were accomplished, and step 5 was considered completed (see Table 1).

### Step 6: Programme Evaluation

A robust evaluation plan was designed prior to implementation, by the project team and academic partners in both the Netherlands and Tanzania. Pertinent data were collected before, during, and after the implementation of the NAA. Both qualitative and quantitative data were collected to enhance triangulation of findings, and perspectives of different stakeholders were planned into the evaluation. Other sources of data (e.g., field notes) were also taken at every meeting in the project, to contribute more context to the interview, focus group, and survey data.

There were several components of the evaluation plan. Firstly, healthcare workers were asked to fill in a questionnaire before and after working with the NAA, containing questions about their experiences working with the NAA, the perceived impact of the NAA on the quality of ANC, and their willingness to incorporate the NAA in their ANC workflow. Secondly, quantitative clinical observations of ANC consultations were undertaken before and after the implementation of the NAA to observe differences in ANC processes. Thirdly, in-depth interviews were held with healthcare workers and pregnant women about their experiences with the NAA. Fourthly, an overall evaluation of the Women Centered Care Project was undertaken to assess progress in contributing to reductions in maternal and neonatal health in the district (39). Results of the evaluation will be presented in future work (in preparation). The memorandum of understanding signed at the beginning of the project did not have a sustainability plan to continue the work of the NAA after the end of the project. This resulted in a termination of working with the NAA during ANC in the participating healthcare facilities as the local government did not have the resources and did not prioritise taking over activities or processes related to the NAA. The three tasks of step 6 related to preparing and conducting the evaluation were fully accomplished and the task related to communicating the results to partners was partly accomplished (see Table 1). Consequently, step 6 of Intervention Mapping was considered completed.

## Discussion

This study applied the planning tool of Godin et al. (35) combined with broader knowledge on Intervention Mapping and semi-structured interviews to evaluate the development and implementation of the NAA, and electronic clinical decision and support system for ANC in Magu district, Tanzania. We found that 22 of the 40 tasks and five of the six steps of Intervention Mapping were completed. Specifically, tasks related to community engagement, adjustment to local context, implementation in the practical context in collaboration with local partners, and rigorous evaluation were scored as accomplished. However, tasks related to identifying theory-based behaviour change methods were scored as not accomplished.

The results of the current study indicate that the primary focus during intervention development and implementation of the NAA was on collaboration with local partners and the adjustment of the intervention to the local circumstances of the target population. The development process lacked focus on the theoretical aspects of behaviour change and intervention development, and how the NAA would contribute to behaviour change among healthcare workers. These results are in line with several other studies on the implementation of digital health interventions in low- and middle-income countries (40–42). These studies emphasise the importance of stakeholder involvement and feedback sessions with the target population and do not mention the need for applying evidence-based theories while developing a digital intervention (40–42). It has been found that interventions often fail to incorporate theories and evidence in the choices they make for developing their intervention (35, 43), indicating a gap between science and practise (43). Prior studies suggest (43–45) that the use of theory, and Intervention Mapping specifically, is considered to be too time-consuming, expensive, and therefore not feasible to use in all circumstances (43–45). In the context of a non-profit organisation or private company, it might not be common practise to include academic components and apply a theoretical approach. In addition, pre-existing knowledge on behaviour change theories is required to successfully identify relevant behaviour change methods and apply them correctly. That is something practitioners are often not trained in (43). Intervention Mapping acknowledges the necessity of working with a planning group that should always include a behaviour change specialist (34). Also, evidence and published literature on the specific case one is developing an intervention for might be lacking in low- and middle-income countries which makes it challenging to follow all steps of Intervention Mapping (44, 46).

Although, the project team did not use theoretical frameworks such as Intervention Mapping as a guide during the development and implementation of the NAA, it managed to take into account the importance of cultural acceptability and tailoring of interventions to local circumstances. During the development and implementation of the NAA, community engagement was seen as the key ingredient of success which is recognised in theoretical frameworks like Intervention Mapping (47) and supported by several studies (44, 48, 49). Intervention Mapping highlights that an intervention is most likely to fail when it is not culturally appropriate (34). As can be seen in the current study, even though no theories on behaviour change or intervention development have been used, five out of six steps of Intervention Mapping have been carried out. Therefore, Intervention Mapping could still be a useful framework to systematically develop and plan interventions in situations in which less behavioural expertise might be available. Especially since Intervention Mapping explains that behaviour change theories apply even outside the context the theory was tested in (34).

The results of the current study also indicate that the initial idea of an electronic clinical decision and support system as the solution for the high maternal death rates in Magu district came from the board of the African Woman Foundation in the Netherlands. Therefore, the question could be raised about how well community involvement has been achieved. During the process of development and implementation, many stakeholder meetings were held to ensure as much involvement and input as possible, however, the type of intervention that was going to be developed was already determined and any new intervention idea or need emerging from the local stakeholders would not be possible to take into account. However, this was not the case since healthcare workers and other stakeholders expressed their enthusiasm and willingness to work with the NAA from the beginning and showed their dedication during the implementation to make the NAA successful. Related to this it is important to mention that different interests were awakened since the opportunity to receive free electronic devices was at play which might have influenced the behaviour and willingness to participate in the project (50). Therefore, it remains unclear whether the NAA was truly culturally appropriate and the best possible solution to solve the problem of maternal death in the district.

Data shows that the NAA intervention lacked a clear programme objective and desired outcomes. These limitations might influence the effectiveness, sustainability, and usefulness of the NAA even more than the lack of reference to behaviour change theories. Essential for developing interventions to improve quality of care, is engaging the broader health system and making stakeholders accountable (51). Stating clear programme goals and sharing expectations about the desired change is part of this. Although, involvement with all stakeholders was abundant and the approval of the District Medical Officer was acknowledged as essential to conduct all activities, the project team of the Women Centered Care Project was responsible for the development and implementation of the NAA. Looking back, it is essential for the sustainability of the project to share ownership and responsibilities with stakeholders and to create clear goals and desired change objectives. In this light, we recommend that the existing government technological infrastructure should also have been taken into account during the development process to make the NAA a better fit within the existing (digital) healthcare structures.

Although, a pre-pilot was conducted in another district unforeseen challenges arose in Magu district. These could have been partly avoided in four ways. First, the pre-pilot could have been conducted with the final version of the NAA instead of the test version. Second, the stakeholder meetings could have focused more on the practical use of the NAA in the healthcare facility and its environment. Third, the choice for the type of device to run the NAA application could have been a choice made by the end-users and not by the Board. And last, a script with potential problems could have been developed before the implementation to anticipate and prepare for potential challenges.

### Strengths and Limitations

The strength of the current study is the practical and accessible description of the evaluation and lessons learned from the development and implementation process of an electronic clinical decision and support system in a low- and middle-income country, which to the best of our knowledge, has not been described before. A limitation of the study is the fact that researchers involved in the evaluation were also involved in the implementation. Although, this allows for a clear understanding of all the documents and processes, it carries a risk of bias in the interpretation of data. Results were verified by two initial developers of the NAA. The reason only two interviews were conducted was based on the dissolution of the Women Centered Care Project, and our inability to contact additional relevant key informants who were part of the project and the development of the NAA. Despite the fact that only two semi-structured interviews were conducted, we believe that the results present a realistic representation of the development and implementation process. Also, as this was a retrospective evaluation, there is a potential risk of recall bias. To reduce this bias, project archives were consulted to check for inconsistencies and search for written proof to base the results on. This project did not have the aim to do a quality assessment of the documents included in the project but used the documents to gain insight into de decision-making process and the involvement of the various stakeholders. Furthermore, locally specified goals and objectives were not taken into account as much as they could be included, which could have impacted ownership of the NAA. In addition, we found that it is crucial to identify the intended behavioural outcome of the intervention as well as the behavioural determinants before intervention development, and rely on theory and empirical research in doing so. Also, more explicit attention could have been given to both adopter and innovation characteristics to promote NAA's implementation and use [see for example (30)]. Despite these limitations, we believe that this study contributes to providing valuable insights for future digital health tool developers in low- and middle-income countries.

## Conclusion

In sum, this retrospective evaluation of the development and implementation process of the NAA revealed that 5 out of 6 steps of Intervention Mapping were successfully completed. Specifically, the tasks related to community engagement, adjustment to local context, implementation in the practical context in collaboration with local partners, and rigorous evaluation were satisfactorily accomplished. Based on the lessons learned during the process of developing and implementing the NAA we recommend future programme developers to (1) engage the community and listen to their insights, (2), focus on clear programme goals and the desired change, (3), consult or involve a behaviour change specialist, and (4), anticipate potential problems in unexpected circumstances. As such, the current description of the development and implementation of an electronic clinical decision and support system, including lessons learned, is unique and contributes to building necessary knowledge to successfully develop, and implement digital health tools in low- and middle-income countries.

## Data Availability Statement

The raw data supporting the conclusions of this article will be made available by the authors, without undue reservation.

## Ethics Statement

Approval for this study was obtained from the National Institute of Medical Research of Tanzania (MR/53/100/103-244-245-349-399) and Maastricht University in the Netherlands (OZL_188_10_02_2018_S32). The patients/participants provided their written informed consent to participate in this study.

## Author Contributions

SP, KM, LS-Z, and LE: conceptualisation and validation. SP, LS-Z, and KM: methodology and writing-original draft preparation. AL and SP: data collection. SP, LE, and AL: analysis. LE, AL, KM, LS-Z, RR, and JW: writing-review and editing. KM, LS-Z, RR, and JW: supervision. SP, LS-Z, and AL: project administration. All authors contributed to the article and approved the submitted version.

## Conflict of Interest

The authors declare that the research was conducted in the absence of any commercial or financial relationships that could be construed as a potential conflict of interest.

## Footnotes

^1^van Pelt S, van der Pijl M, Shields-Zeeman L, de Wit J, Ruiter R, Massar K. Women's perceptions of antenatal care and digital health tools to improve healthcare: A qualitative study in Magu District, Tanzania. (Under review).
